# Supplementary material for: Environmental and Social Factors Associated with High Chronic Kidney Disease Mortality Rates in Municipalities of Guatemala: An Ecological Study of Municipal-Level Mortality Data
Source: Int J Environ Res Public Health. 2023 Apr 17;20(8):5532. doi: 10.3390/ijerph20085532 (PMC10198324; doi:10.3390/ijerph20085532)
Supplement: Supplementary file 1 [file ijerph-20-05532-s001.zip › Supplementary file 2-IJERPH.docx]

Alejandro Cerón “Environmental and social factors associated with high chronic kidney disease mortality rates in municipalities of Guatemala”

Supplementary table 1. Bivariate analysis. Logistic regression results for each variable and population group (alpha 0.05).

|  | **Población total** | | **Población total masculina** | | **Población total femenina** | | **Población menor de 60** | | **Población menor de 45** | | **Población menor de 20** | |
| --- | --- | --- | --- | --- | --- | --- | --- | --- | --- | --- | --- | --- |
| **Variable** | **Coeficiente** | **P>\|t\|** | **Coeficiente** | **P>\|t\|** | **Coeficiente** | **P>\|t\|** | **Coeficiente** | **P>\|t\|** | **Coeficiente** | **P>\|t\|** | **Coeficiente** | **P>\|t\|** |
| Temperatura promedio | 4.115534 | 0.000 | 5.902844 | 0.000 | 2.385239 | 0.000 | 4.520553 | 0.000 | 2.032576 | 0.000 | 0.4297609 | 0.042 |
| Altitud | -0.0235651 | 0.000 | -0.0343793 | 0.000 | -0.0131491 | 0.000 | -0.025862 | 0.000 | -0.0116369 | 0.000 | -0.0020999 | 0.088 |
| Pobreza (I) | 4.96665 | 0.000 | 5.879775 | 0.004 | 4.101405 | 0.000 | 5.689922 | 0.000 | 2.173423 | 0.011 | 0.9442139 | 0.047 |
| Fuerza laboral en agricultura (E) | -1.752626 | 0.187 | -1.598 | 0.404 | -1.915892 | 0.061 | -2.019036 | 0.163 | -0.1911477 | 0.812 | -0.175536 | 0.695 |
| Superficie de uso agrícola (E) | 1.851275 | 0.553 | -0.8094134 | 0.857 | 4.397746 | 0.066 | 1.986589 | 0.559 | -0.3459142 | 0.854 | -0.7220312 | 0.492 |
| Intensidad de uso de la tierra (I) | -10.47643 | 0.002 | -18.85728 | 0.000 | -2.503932 | 0.336 | -11.65679 | 0.001 | -4.144381 | 0.042 | 1.30839 | 0.250 |
| Tasa unidades salud primer nivel | -0.1725139 | 0.680 | 0.2430416 | 0.668 | -0.5688097 | 0.119 | -0.1580616 | 0.730 | -0.1208718 | 0.610 | -0.0672303 | 0.595 |
| Tasa unidades salud totales | 0.0439663 | 0.912 | 0.4013836 | 0.455 | -0.2978864 | 0.393 | 0.079105 | 0.856 | 0.0316943 | 0.888 | 0.0085366 | 0.943 |
| Maya porcentaje | -38.63164 | 0.000 | -52.62828 | 0.000 | -25.17045 | 0.000 | -43.62232 | 0.000 | -20.29701 | 0.000 | -6.899856 | 0.006 |
| Ladino porcentaje | 39.86705 | 0.000 | 55.76921 | 0.000 | 24.55423 | 0.000 | 45.01093 | 0.000 | 20.83431 | 0.000 | 6.233635 | 0.016 |
| IARNA territorio 01 | -26.64688 | 0.001 | -36.78909 | 0.002 | -16.88279 | 0.003 | -29.98727 | 0.000 | -11.98444 | 0.012 | -3.219259 | 0.209 |
| IARNA territorio 02 | -42.22027 | 0.000 | -52.27715 | 0.004 | -32.5542 | 0.000 | -47.00752 | 0.000 | -21.10599 | 0.003 | -7.552473 | 0.052 |
| IARNA territorio 03 | 12.42719 | 0.443 | 14.80447 | 0.539 | 10.34306 | 0.375 | 13.39696 | 0.447 | 3.226294 | 0.740 | -3.715677 | 0.477 |
| IARNA territorio 04 | 35.96238 | 0.000 | 47.37569 | 0.001 | 24.71418 | 0.000 | 39.64467 | 0.000 | 23.06338 | 0.000 | 5.40246 | 0.080 |
| IARNA territorio 05 | -8.100908 | 0.334 | -15.00312 | 0.229 | -1.376792 | 0.820 | -7.072897 | 0.439 | -3.571697 | 0.478 | 5.31548 | 0.049 |
| IARNA territorio 06 | -11.28789 | 0.351 | -24.72613 | 0.170 | 1.334906 | 0.878 | -12.85009 | 0.330 | -8.010199 | 0.270 | -1.703078 | 0.663 |
| IARNA territorio 07 | 51.08626 | 0.000 | 81.15904 | 0.000 | 22.20329 | 0.001 | 55.14631 | 0.000 | 24.15232 | 0.000 | 1.675454 | 0.564 |
| IARNA territorio 08 | -43.81173 | 0.021 | -56.09569 | 0.047 | -31.7158 | 0.020 | -48.4263 | 0.019 | -22.78345 | 0.046 | -9.707305 | 0.113 |
| IARNA territorio 09 | -7.661397 | 0.469 | -13.76462 | 0.382 | -1.661206 | 0.828 | -8.131019 | 0.481 | -8.027886 | 0.206 | -1.445194 | 0.672 |
| IARNA territorio 10 | 26.35791 | 0.444 | 30.23566 | 0.555 | 23.19354 | 0.349 | 31.78885 | 0.396 | 14.63582 | 0.479 | 15.13936 | 0.172 |
